# Laser-scanning velocimetry: A confocal microscopy method for quantitative measurement of cardiovascular performance in zebrafish embryos and larvae

**DOI:** 10.1186/1472-6750-7-40

**Published:** 2007-07-10

**Authors:** Michael H Malone, Noah Sciaky, Lisa Stalheim, Klaus M Hahn, Elwood Linney, Gary L Johnson

**Affiliations:** 1 Department of Pharmacology and Lineberger Comprehensive Cancer Center, University of North Carolina at Chapel Hill, Chapel Hill, NC 27599, USA; 2 Department of Molecular Genetics and Microbiology, Duke University, Durham, NC 27708, USA

## Abstract

**Background:**

The zebrafish *Danio rerio *is an important model system for drug discovery and to study cardiovascular development. Using a laser-scanning confocal microscope, we have developed a non-invasive method of measuring cardiac performance in zebrafish embryos and larvae that obtains cardiovascular parameters similar to those obtained using Doppler echocardiography in mammals. A laser scan line placed parallel to the path of blood in the dorsal aorta measures blood cell velocity, from which cardiac output and indices of vascular resistance and contractility are calculated.

**Results:**

This technique, called laser-scanning velocimetry, was used to quantify the effects of pharmacological, developmental, and genetic modifiers of cardiac function. Laser-scanning velocimetry was applied to analyze the cardiovascular effects of morpholino knockdown of osmosensing scaffold for MEKK3 (OSM), which when mutated causes the human vascular disease cerebral cavernous malformations. OSM-deficient embryos had a constricted aortic arch and markedly increased peak cell velocity, a characteristic indicator of aortic stenosis.

**Conclusion:**

These data validate laser-scanning velocimetry as a quantitative tool to measure cardiovascular performance for pharmacological and genetic analysis in zebrafish, which requires no specialized equipment other than a laser-scanning confocal microscope.

## Background

The zebrafish *Danio rerio *are widely used in genome-wide screens to identify genes that regulate the development and function of the vertebrate cardiovascular system [1-3]. Zebrafish are powerful model organisms to define genes relevant to human cardiovascular disease because of their similarity in genetic complexity to mammals, the ease of selective gene knockdown, and their suitability for large-scale mutagenesis screens [4,5]. A beating heart and functional circulatory system forms within 26 hours of fertilization, and within 48 hours the heart develops into a two-chambered conformation, complete with functional valves. Because of their transparency, severe morphological abnormalities in cardiac development are easily observed, but the functional consequences of these defects are difficult to quantify. Methods are needed for functional analysis of cardiovascular defects that can be utilized by any investigator studying zebrafish cardiovascular physiology. Using an unmodified, commercially-available laser-scanning confocal microscope, we have developed a novel, non-invasive method of measuring cardiac performance in zebrafish embryos that provides measurements similar to those obtained with Doppler echocardiography. The technique, called laser-scanning velocimetry provides a continuous measurement of blood cell velocity that is necessary for estimating cardiac output (velocity integral) and measuring indices of vascular resistance (peak deceleration) and ventricular contractility (peak acceleration).

We first demonstrate that laser-scanning velocimetry is a sensitive and precise tool for measuring the physiological changes during normal cardiovascular development and drug-induced cardiotoxicity. We then use laser-scanning velocimetry to evaluate the cardiovascular function of zebrafish embryos with reduced expression of osmosensing scaffold for MEKK3 (OSM) – a scaffold protein, whose mutation in humans leads to the development of a vascular disorder called cerebral cavernous malformations [6]. Using microangiography we demonstrate that OSM morphant embryos have a constricted aortic arch. Analysis by laser-scanning velocimetry functionally verified the presence of aortic stenosis through a markedly increased peak cellular velocity. These experiments establish laser-scanning velocimetry as a simple yet powerful technique that allows quantitative physiological analysis of cardiac performance in zebrafish embryos.

## Results and discussion

### Linescan images depict cell flux and velocity within blood vessels

**Figure 1:**
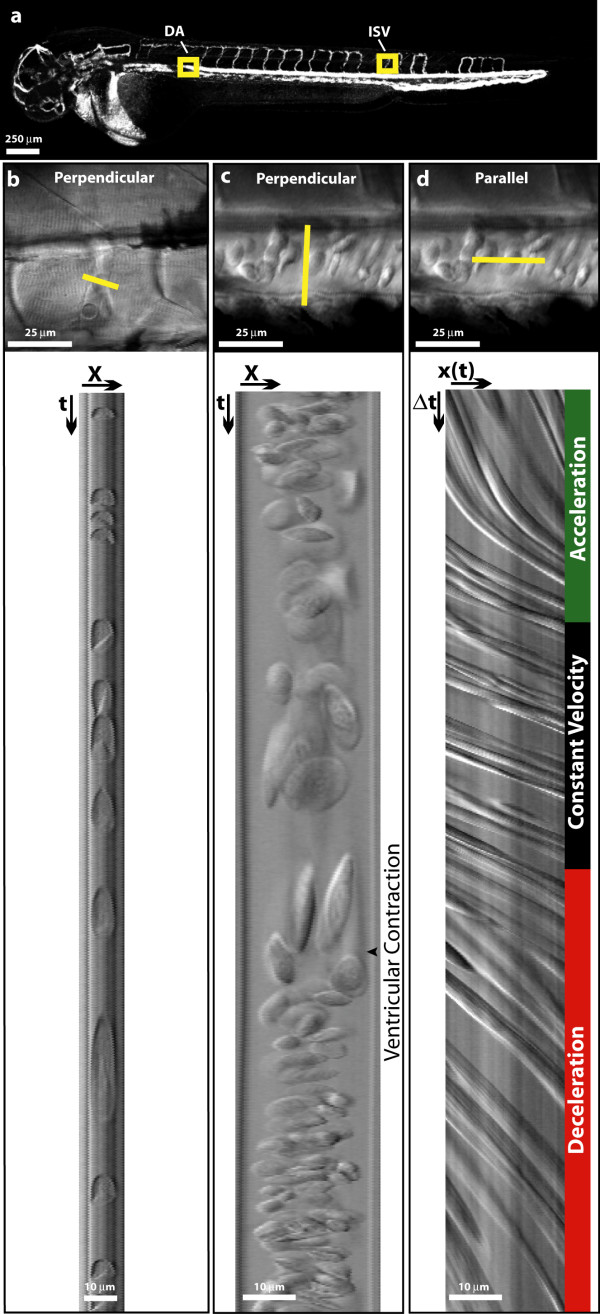
**Acquring linescan images from *Danio rerio *embryos**. (a) *In silico *microangiogram of a 48 hpf wild-type zebrafish embryo. Regions of interest used for acquiring linescan images are boxed (intersomitic vessel = ISV, dorsal aorta = DA). (b and c) A single plane (XY) image (Top) showing the orientation of scan lines used to create perpendicular linescans (Bottom) of the intersomitic vessels (b) and dorsal aorta (c). The boundary between slow moving (elongated) cells and fast moving (compressed) cells reflects a ventricular contraction and is easily observed in the Dorsal Aorta (c). (d) A parallel linescan acquired from the dorsal aorta shows a series of lines whose slope is inversely proportional to cellular velocity. Distinct regions of acceleration, constant velocity, and deceleration are observed.

Linescans are images constructed with laser-scanning microscopes by repeatedly imaging a one pixel-wide line. Whereas traditional two-dimensional images provide spatial information in both the x- and y-dimensions, linescans contain spatial information in the x-dimension and temporal information in the y-dimension. When the scan line is placed across a vessel, either perpendicular or parallel to the flow of blood, the resulting linescan images contains information about the dynamics of circulation (Figure 1). Because images of the linear region of interest can be acquired rapidly, linescans provide excellent temporal resolution. In a perpendicular linescan image, the width of a blood cell in the x-dimension of the image is proportional to the physical width of the cell, whereas the length is proportional to the time required for the cell to cross the imaging line (Figure 1b and 1c). Faster moving cells cross the scan line more quickly and appear to be shorter than slower moving cells. In vessels with high cell flux, such as the dorsal aorta, periodic changes in apparent cell length are evident and reflect the periodic change in cellular velocity caused by ventricular contraction and subsequent relaxation (Figure 1c). Cell flux can easily be determined from perpendicular linescans by simply counting the number of cells that completely cross the imaging line in a given unit of time.

Many diagnostic indices of cardiac function can be calculated from a continuous measurement of the velocity of blood cells flowing through the dorsal aorta (Table 1). While cellular velocity can be calculated from the length of a cell in a perpendicular linescan image, cell lengths are difficult to measure within high-flux vessels, and the velocity calculation assumes that the cells are all spherical and have the same dimensions. Furthermore, velocity calculations based on perpendicular linescans are discontinuous: because each measurement represents the average velocity for the duration of time the cell was crossing the scan line, the instantaneous velocity of a cell is not attainable. These limitations are overcome using a scan line placed parallel to the flow of blood (Figure 1d). Parallel linescans acquired from the dorsal aorta contain a series of lines whose slopes alternate in a sigmoidal fashion (Figure 1d, bottom panel). These lines are created when a high-contrast cellular feature (such as the edge of a cell) crosses the scan line. Each scan captures the position of these features along the line. Successively adding these single line images together creates a linescan image where the positions of these features in the x-dimension are recorded as a function of time in the y-dimension. If a cell is stationary, its position will not change along the line and it will appear as a vertical stripe, whereas a cell moving faster than the scan speed will appear as a horizontal streak. Thus velocity is inversely proportional to the slope of the line traced by the cell in the linescan. Three phases of cell traces can be observed in most parallel linescans: convex, linear, and concave, which correspond to phases of accelerating, constant and decelerating velocity, respectively. The continuity of velocity information provided by laser-scanning velocimetry makes it possible to measure blood acceleration and deceleration – parameters previously only attainable using Doppler echocardiography – that are indices of vascular resistance and contractility [7-9].

**Table 1:** Measured quantities, their physiological correlates and diagnostic utility

**Physical Measurement**	**Physiological Parameter**	**Diagnostic Relevance**
Peak Velocity	Peak Velocity	↑ in Aortic Stenosis, ↓ in Heart Failure
Minimum Velocity	Minimum Velocity	Negative with valvular dysfunction
Peak Acceleration	Contractility	↓ in Heart Failure
Peak Deceleration	Peripheral Resistance	Indicative of vascular tone and development
Velocity Integral	Stroke Volume and Cardiac Output	↓ in Heart Failure

A scan line placed in the middle of the vessel was found to be optimal for velocimetry measurements because the flux of cells crossing the line is much higher than at the extreme periphery (Figure 1c, bottom panel). While blood flow within this long artery should be laminar, significant differences in cellular velocity were not observed as the scan line was moved from the center of the lumen to the periphery. The maximum cellular velocity obtained from a scan line placed in the middle of the lumen of a 48 hpf embryo was 0.2459 ± 0.0102 cm/s, and that obtained from a scan line at the periphery of cell flux was 0.2455 ± 0.0276 cm/s. The apparently constant flow profile may be explained by the requirement of cell motion for laser-scanning velocimetry to measure velocities. While the plasma velocity may be lower very near the vessel wall, velocities in this region cannot be determined by laser-scanning velocimetry because few cells are present (Figure 1c, bottom panel).

### Laser-scanning velocimetry is a sensitive technique for assessing drug-induced cardiotoxicity

**Figure 2:**
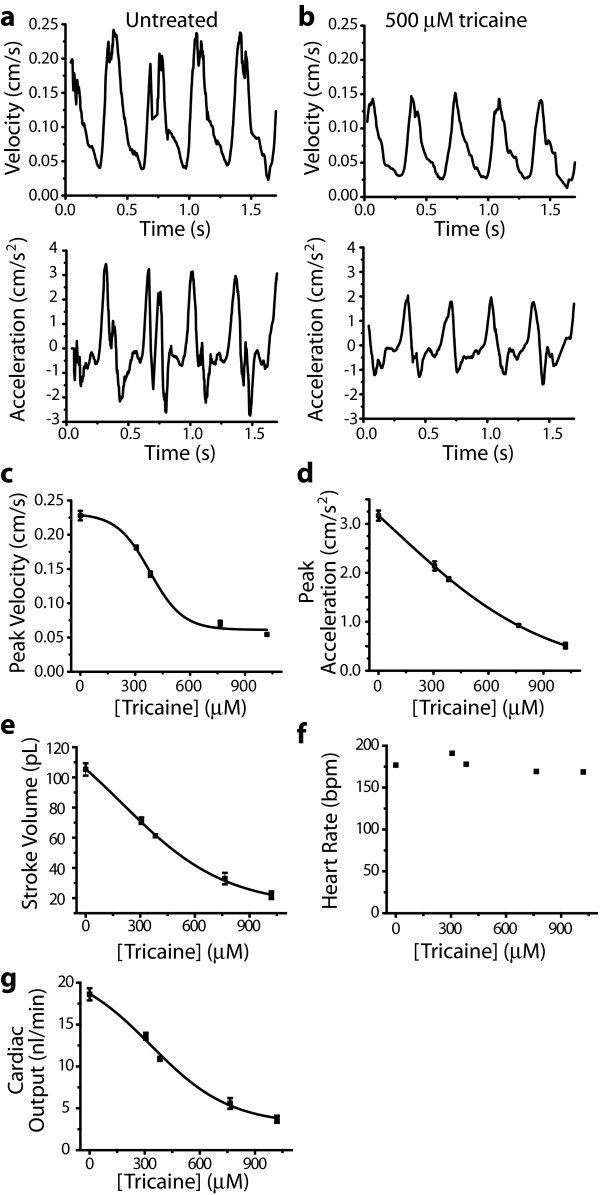
**Laser-scanning velocimetry measures the cardiac toxicity of tricaine**. (a and b) Velocity and acceleration profiles acquired from the dorsal aorta of 48 hpf embryos untreated (a) or treated (b) with 500 μM tricaine. (c-g) Cardiac parameters of a single *Danio rerio *embryo treated with successively increasing doses of tricaine. Dose-response relationship of peak velocity (c), peak acceleration (d), stroke volume (e), heart rate (f) and cardiac output (g). Error bars represent the SEM of values obtained from four to six heartbeats in a single embryo. Lines are a non-linear least squares fit to a sigmoidal dose-response curve. Laser-Scanning Velocimetry Data Analyzer smoothing parameters used for trace, velocity, and acceleration smoothing were 5, 60, and 80, respectively.

To demonstrate that laser-scanning velocimetry is a sensitive technique capable of measuring subtle differences in cardiac performance caused by pharmacological modification, we acquired linescans from a two day old zebrafish embryo successively treated with increasing concentrations of the aquatic anaesthetic tricaine, which is known to suppress cardiac function at high doses. Linescans obtained from untreated embryos and those bathed in a cardiac-suppressing dose of tricaine (500 μM) yielded velocity-versus-time profiles similar to those obtained by continuous-wave Doppler echocardiography in mammals (Figure 2a and 2b). The tricaine treatment caused a 40% reduction of peak cell velocity from 0.25 cm/s to 0.15 cm/s (Figure 2a and 2b). As the concentration of tricaine increased, peak velocity decreased with a sigmoidal dose-response relationship (Figure 2c). It has long been appreciated in Doppler echocardiography that peak acceleration is closely correlated with ventricular contractility [7,10]. Acceleration of blood cells was determined from the derivative of the velocity-versus-time profiles, and was also decreased by tricaine (Figure 2a, b, and 2d).

Stroke volume, the volume of blood ejected by the heart in one beat, can be determined by integrating the continuous velocity versus time graphs obtained by laser-scanning velocimetry and multiplying by the cross-sectional area of the aorta. Since the heart is a pump, stroke volume is an excellent functional measure of cardiac performance. Estimates of stroke volume decreased significantly over a concentration range of tricaine that caused little change in heart rate (Figure 2e and 2f). Heart rates were found to remain relatively steady at low to moderate doses of tricaine (Figure 2f). Because heart rates varied little over the dose range tested, cardiac output decreased proportionately with decreases in stroke volume (Figure 2e and 2g). Our assessment of cardiac output by laser-scanning velocimetry (~ 18.6 nL/min at 48 hpf) is in good agreement with volume-approximating methods of estimating cardiac output but our method is based on actual output and does not assume normal heart morphology [11,12].

Absolute measurements of stroke volume can be obtained when the scan line is placed along the outflow tract prior to any arterial branches. While it is not always possible to image the outflow tract prior to arterial branching, an estimate of stroke volume can be obtained by measuring cellular velocities along the dorsal aorta immediately caudal to the fusion of the lateral dorsal aortas (Figure 1a). Relative comparisons of stroke volume and cardiac output can be made between embryos of the same developmental stage when the scan line is placed in the same position along the artery. To investigate the dependence of stroke volume and cardiac output estimates on scan line position, measurements were acquired along the dorsal aorta in a rostral location (immediately caudal to the fusion of the lateral dorsal aortas) a caudal location (above the cloaca). When the scan line is placed in the caudal position, peak velocity and acceleration are underestimated by 15–20% (see Additional file 1, panels a-c). Interestingly, stroke volume and cardiac output estimates are not significantly changed when measured with either a rostral or a caudal scan line position (see Additional file 1, panels d and e).

### Correlating structure and function in the developing heart

**Figure 3:**
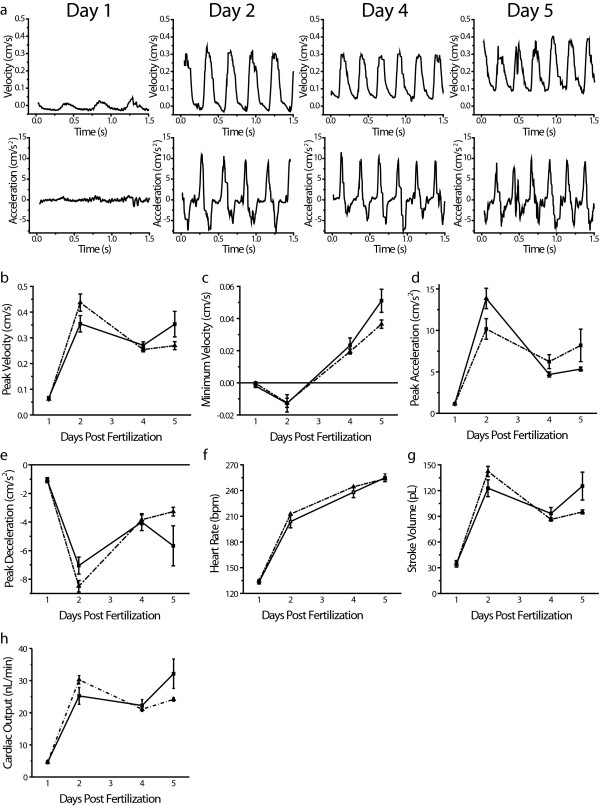
**Functional assessment of cardiac performance in the developing zebrafish heart**.(a) Representative velocimetry and acceleration profiles from *Danio rerio *embryos and larvae during development. (b-h) Cardiovascular parameters measured at 1, 2, 4 and 5 days post-fertilization from a single zebrafish (dotted line with triangles) and a population average (solid line with squares). (b) Peak velocity, (c) minimum velocity, (d) peak acceleration, (e) peak deceleration, (f) heart rate (no error bars are present for the single fish measurement because beat-to-beat variability is not calculated), (g) stroke volume, and (h) cardiac output. Single-fish data presented are the average obtained by analyzing four to six individual heart beats. Error bars are the SEM and represent intrafish variability. For population average data n = 5 for days 1, 2, and 4; n = 4 for day 5. Error bars are the SEM and represent interfish variability. Laser-Scanning Velocimetry Data Analyzer smoothing parameters used for trace, velocity, and acceleration smoothing were 5, 10, and 20, respectively.

The two-chambered heart of *Danio rerio *matures into an adult-like morphology by five days post fertilization (dpf). We used laser-scanning velocimetry to correlate differences in cardiac function with developmental changes in heart structure (Figure 3). Because laser-scanning velocimetry is non-invasive, the development of individual embryos can be followed over time. Representative velocimetry profiles from zebrafish at 1, 2, 4, and 5 days post fertilization are shown in Figure 3a. Changes in cardiovascular parameters between days 1 and 5 are shown for both a single fish and a population (Figure 3b–h). Trends in the development of cardiovascular function observed in individuals were representative of the changes observed in the population, indicating the legitimacy and reproducibility of laser-scanning velocimetry.

At day one, the zebrafish heart exists as an elongated, primitive tube that regularly contracts but has not yet developed into a two-chambered heart [13]. Blood is ejected from the one day heart with gradual but rather sustained changes in velocity (Figure 3a). Both acceleration and deceleration are minimal at this stage (Figure 3d and 3e). By day two the heart has differentiated into a two-chambered heart, has looped into its adult conformation, and the atrio-ventricular valves have formed. Velocity-versus-time peaks for each heart beat are sharper and more frequent than at day one (Figure 3a). Notably, by day two there are significant increases in peak velocity and peak acceleration, as well as in estimates of stroke volume, and cardiac output (Figure 3b, d, g, and 3h). Peak deceleration is an index of vascular resistance and is greatest at this time. This increase in resistance provides enough opposing force to the forward motion of blood that some retrograde motion, indicated by negative minimum velocity values, is observed (Figure 3c and 3e). After day two, vascular resistance decreases and minimum velocities are greater than zero (Figure 3c and 3e). Because there is no reversal of flow direction, peak acceleration actually decreases between days two and three. Thus, it should be noted that, as in ultrasound, peak acceleration is a load-dependent measurement and should be used as an index of contractility only when there are no significant differences in vascular resistance.

Between days 2 and 5 changes in heart chamber orientation and differentiation continue, but no overt structural changes occur [13]. This is reflected by only modest changes in peak velocity, stroke volume, and cardiac output between days two and five (Figure 3b–e, and 3g).

### Laser-scanning velocimetry provides functional evidence for the presence of an aortic constriction in OSM-deficient embryos

**Figure 4:**
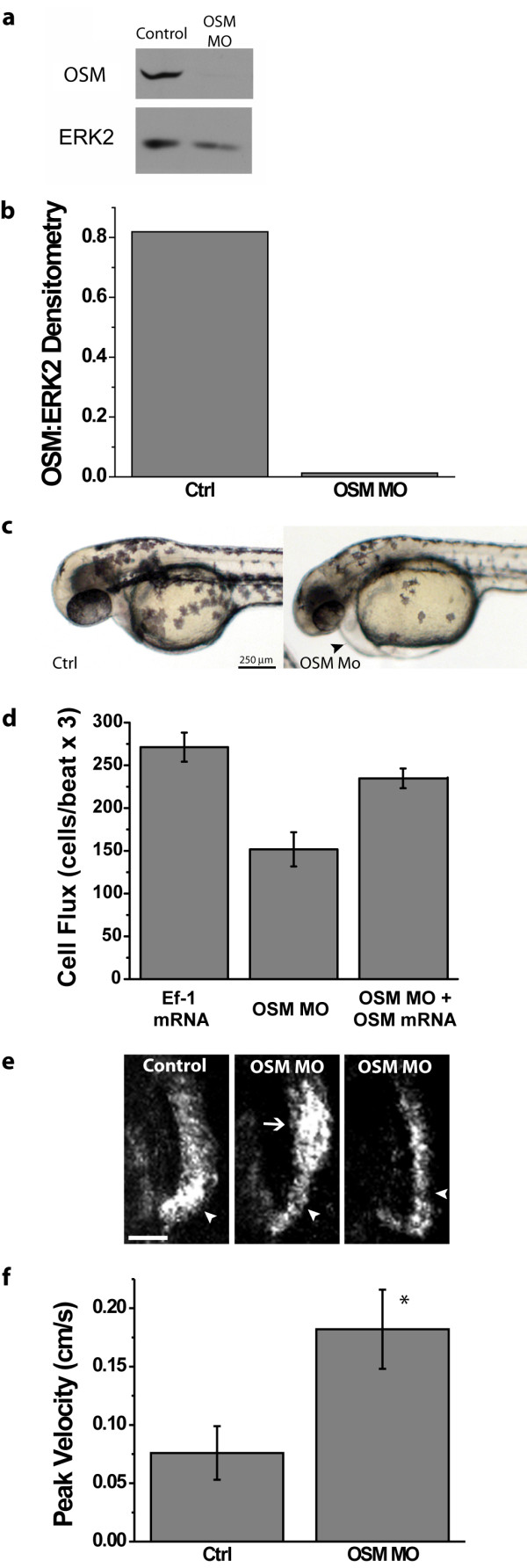
**Laser-scanning velocimetry provides functional evidence for the presence of an aortic arch constriction in OSM-deficient embryos**. (a) Western blotting for OSM in lysates from control or OSM morpholino-injected embryos harvested at 48 hpf shows nearly complete repression of OSM expression. The abundance of ERK2 is shown as a loading control. (b) Intensity of OSM bands normalized to ERK2 by densitometry for western blots shown in Panel a. (c) OSM morphant embryos at 48 hpf exhibited pericardial edema (arrowhead). (d) Co-injection of OSM mRNA along with OSM morpholino restored cell flux to levels similar to that of control embryos. Cell flux was quantified by scan lines drawn perpendicular to the dorsal aorta above the cloaca. Error bars represent the SEM, n = 3. (e) *In silico *microangiography of the aortic arch (arrowheads) at 28 hpf shows constricted flow through this region of the outflow tract in OSM-deficient embryos. A region of post-stenotic dilation (arrow), the result of turbulent flow, is occasionally observed. Scale bar is 25 μm. (f) Peak blood cell velocity, measured by laser-scanning velocimetry is increased in OSM-deficient embryos. Error bars represent SEM. Asterisk denotes p = 0.0017 for the two-tailed Student's T-test, n = 3. OSM morpholino #1 was used for the data presented here. Laser-Scanning Velocimetry Data Analyzer smoothing parameters used for trace, velocity, and acceleration smoothing were 5, 10, and 20, respectively.

It was recently shown that *valentine *mutant embryos have a mutation at the *ccm2*/OSM locus that prevents concentric patterning of the myocardium and results in a loss of blood flow. To demonstrate that laser-scanning velocimetry is a valuable diagnostic tool to study the functional consequences of genetic modification of heart development, we studied the cardiovascular phenotype of embryos where expression of the *ccm2 *gene product OSM was inhibited by morpholino suppression of translation. Translation-blocking morpholino oligonucleotides targeted at or near the translation start site of the *Danio rerio ccm2*/*osm *mRNA were injected into embryos between the 1 and 4 cell stages. OSM protein expression in morpholino-injected embryos was reduced to nearly undetectable levels as demonstrated by western blotting at 48 hpf (Figure 4a and 4b). When normalized to an ERK2 loading control by densitometry, OSM expression was reduced by 98% relative to control embryos (Figure 4b). OSM-deficient embryos had pericardial edema (Figure 4c, arrow), and significantly reduced circulation of erythrocytes. Each of two OSM morpholinos generated the same phenotypes with slightly different potencies (please see Additional file 2). To show that the reduced circulation was specific for the repression of *ccm2*/*osm *translation, we rescued the morphant phenotype by co-injecting *in vitro *transcribed mRNA from the entire coding region of *Danio rerio ccm2/osm *cDNA along with the morpholino designed to target the 5' untranslated region of the endogenous *ccm2*/*osm *mRNA (OSM MO#1). Quantification of cell flux using perpendicular linescan images indicated that erythrocyte flux in OSM-deficient embryos was reduced to 55% that of control embryos, and this phenotype was almost completely restored by co-injection of *ccm2*/*osm *mRNA (Figure 4d)

Because homozygous null mice for *ccm1*/Krit1 die at E8.5 due to a constricted aortic arch [14], we investigated whether the decreased circulation in OSM morphants was due to defective vessel formation. *In silico *microangiography was used to outline the functional vasculature in control and OSM morphant embryos. *In silico *microangiography is a simple and non-invasive technique that uses digital image processing of images obtained using widefield microscopy and a CCD camera, and delivers results similar to laborious dye injection-based microangiography methods [15]. Microangiograms of the outflow tract of 26 hpf embryos revealed constricted flow through the aortic arch of morphant but not control embryos (Figure 4e). Movies of the aortic arch clearly show erythrocytes travelling side-by-side through the arch of a control embryo while there is only room for single-file travel through an OSM-deficient embryo (please see Additional file 3). Vessel patterning and lumen formation was unaffected in other vessels (please see Additional file 2), indicating a specific defect in aortic arch patterning.

In human and veterinary cardiology, Doppler echocardiography is used to diagnose aortic stenosis (constrictions). One characteristic change observed in patients with aortic stenosis is an increase in peak velocity because the constricted passage acts as a nozzle to increase the velocity of blood flowing through it [16]. Laser-scanning velocimetry measurements indicated that the peak cellular velocity in OSM morphants was 0.182 ± 0.019 cm/s versus 0.076 ± 0.011 cm/s in control embryos (Figure 4f). These results demonstrate that laser-scanning velocimetry can be used as a diagnostic tool for studying the genetics of cardiac performance as well as identifying genes responsible for congenital heart diseases.

## Conclusion

Despite the widespread use of zebrafish for understanding cardiac development and function, few quantitative tools exist to measure functional cardiovascular phenotypes in embryos. Laser-scanning velocimetry meets this need, using a standard laser-scanning confocal microscope for non-invasive measures of cardiac function. High-resolution ultrasound has been used to measure cardiovascular performance in adult zebrafish [17], and technically could be applied to the analysis of the zebrafish embryonic cardiovascular system. Unlike confocal microscopes that are available at many research institutions, high-resolution ultrasound requires expensive and highly specialized equipment that are not generally available to many investigators. We have shown that laser-scanning velocimetry provides sensitive and precise measures of cardiac function including contractility, cardiac output, vascular resistance, heart rate, and stroke volume. Our data demonstrate that laser-scanning velocimetry easily measures drug-induced cardiotoxicity, performance changes during cardiovascular development and can diagnose anatomic lesions such as aortic stenosis in genetically-manipulated embryos.

Laser-scanning velocimetry provides quantitative and precise measures of several indices of cardiac performance (summarized in Table 1) and offers significant improvements over existing pre-clinical cardiovascular diagnostic techniques. A recent report by Pan, et. al. combines linescans with correlation spectroscopy to measure the velocity of cells travelling through the dorsal aorta of embryonic zebrafish [18]. While determining velocity vectors by correlation spectroscopy is a significant advance, this technique requires two sequential measurements that severely limit its temporal resolution. Discontinuous measurements of cellular velocities have been made in zebrafish embryos using video microscopy, and in transgenic mouse embryos expressing GFP-labeled erythrocytes using perpendicular linescans [15,19]. Dual spot cross correlation methods of measuring blood cell velocity have existed since the mid-1970s and should be applicable to measuring blood cell velocity in zebrafish embryos with good temporal resolution [20-23]. These methods use two photodiodes spaced a fixed distance apart along the path of blood flow, and cell velocities are calculated based on the time required for a cell to pass between the two detectors. Because cross-correlation methods only measure the average speed of a cell as it passes between the two detectors, they do not continuously measure velocity. This continuity is required for calculating acceleration and deceleration, which contains important information about contractility and peripheral resistance. The use of a parallel scan line allows us to continuously measure the velocity of the same object so long as it is located within the linear scanning region. This feature is what makes laser-scanning velocimetry unique in its temporal resolution for both velocity and acceleration measurements.

Video microscopy has also been used to measure cardiac output in *Xenopus laevis, Gallus gallus*, and *Danio rerio *embryos [11,12,24-26]. This technique estimates the volume of blood ejected from the ventricle based on the observed changes in its diameter. Our measurements of cardiac output using laser-scanning velocimetry are in good agreement with these published values [12]. While the cardiac output measurements described here are estimates that assume a circular vessel cross-section, and can be underestimated if linescans are obtained from the aorta after arterial branches, they do not require normal heart morphology and valve function. A more exact determination of cardiac output may be obtained using laser-scanning velocimetry in transgenic zebrafish animals where the vessel dimensions are delineated by an endothelial expressed GFP such as *fli1*-GFP [27].

Recently, the genes responsible for two mutant zebrafish embryos that have similar cardiovascular phenotypes, *santa *and *valentine*, were mapped to *ccm1 *and *ccm2*, respectively [28]. The *valentine *mutant contains a mutation in OSM and exhibits pericardial edema, a dilated heart, and a complete lack of blood flow [28]. Morpholino knockdown of OSM resulted in significantly reduced circulation relative to control zebrafish. The fact that morpholino knockdown of OSM did not completely inhibit blood flow is probably a result of low level OSM expression in contrast to complete loss of functional OSM protein in the *valentine *mutant.

The observation that OSM plays a role in aortic arch lumen formation has important implications for the pathobiology of cerebral cavernous malformations. Mice homozygous for a gene-trap insertion of β-galactosidase into the *ccm1*/Krit1 locus also have a constricted aortic arch [14], indicating that a loss of Krit1 in mouse phenocopies a loss of OSM in zebrafish. When viewed in light of evidence that OSM and Krit1 physically associate with each other at sites of actin reorganization [29], these data suggest that Krit1 and OSM may act as a complex to organize developmental signals required for proper aortic arch lumen formation.

Zebrafish are a popular model for drug screens because of their genetic similarity to humans, the speed of screening in a physiological context and cost-effectiveness [30]. In addition to providing a diagnostic tool for molecular geneticists, laser-scanning velocimetry has obvious applications in pre-clinical drug development. For example, laser-scanning velocimetry could easily be used to identify anti-arhythmic compounds capable of reversing the fibrillating phenotype of the *tremblor *or *reggae *mutants [31]. Similarly, the potency and efficacy of potential inotropic drugs, which increase the force of ventricular contractions in heart failure, could easily be screened in wild-type zebrafish embryos to find those compounds that best increase the peak acceleration of blood cells. As demonstrated in Figure 2, in addition to supporting pre-clinical efficacy studies, laser-scanning velocimetry can also be used to readily identify molecules that possess cardiotoxicity.

Laser-scanning velocimetry quantifies the effects of genetic and pharmacological modifiers of cardiovascular function. With the increasing popularity of zebrafish in basic science and the pharmaceutical industry, the development of additional techniques to correlate physiological function with genes and their chemical modifiers promises to accelerate drug discovery and development in the post-genomic area.

## Methods

### Zebrafish husbandry

Zebrafish (*Danio rerio*) embryos used for velocimetry measurements and morpholino injections were obtained by natural spawning of a wild-type AB* line. Embryos were raised at 28.5°C in 30% Danieau solution(17 mM NaCl, 0.21 mM KCl, 0.12 mM MgSO_4_, 0.18 mM Ca(NO_3_)_2_, 1.5 mM HEPES pH 7.6). Zebrafish were handled in accordance with IACUC approved protocols.

### Laser-scanning velocimetry

Embryos for imaging were placed in a 4-well coverglass-bottom chamber slide (Lab-Tek) in 30% Danieau solution containing the anaesthetic tricaine at a final concentration of 100 μg/ml or as indicated. After a three-minute delay to ensure a steady-state level of anaesthesia, images were captured using an Olympus Fluoview FV1000 laser-scanning microscope with an Olympus PlanApo N (60 ×/1.42 oil) lens using DIC optics. Scan lines were oriented parallel to the flow of blood within the dorsal aorta, just above the yolk ball. Approximately 15,000 lines were acquired per sample at a rate of 0.488 ms/line allowing a maximum velocity of 4.10 cm/s to be measured. Using a 20 μm line and the optical system described above, a minimum scan rate of 8 ms/line is required to record the maximum cellular velocity of 0.25 cm/s observed in these experiments. Cell paths within parallel linescans were traced using the NeuronJ plugin v.1.1.0 for ImageJ v.1.37 [32]. High-quality DIC images and proper adjustment of NeuronJ parameters were critical to obtaining quality tracings. Good results were obtained with the following NeuronJ settings: Hessian smoothing scale = 1.0, cost weight factor = 0.7, snap window size = 1 × 1, path-search window size = 800 × 800, tracing smoothing range = 10, tracing subsampling factor = 1, and line width = 1. NeuronJ trace coordinates were exported as text files then processed to calculate average velocity and acceleration versus time data with Laser-Scanning Velocimetry Data Analyzer software we developed using Visual Basic v6.0 and Access 2003 (Microsoft). Briefly, the position versus time data exported from NeuronJ was smoothed using a moving average function. The instantaneous slope of this smoothed trace was determined for each time point. Where multiple traces were measured for a single point in time, the calculated velocities were averaged. Acceleration data were obtained from the instantaneous slope of velocity-versus-time profiles. A moving average function was used throughout the calculation workflow to minimize noise. The interval of the moving average can be specified as smoothing factors in the data analyzer software. Higher smoothing factors will underestimate measured values but will increase precision. Data are only comparable when calculated using the same smoothing factors and have been specified in the figure legends.

### Tricaine dose-response treatment

A single zebrafish embryo at 30 hpf was placed in a coverglass-bottom chamber slide containing 30% Danieau solution without tricaine. Parallel linescans were acquired from the free-floating, non-anesthetized embryo to obtain baseline values of an untreated embryo. After a baseline image was collected, the 30% Danieau was sequentially replaced with 30% Danieau solutions containing increasing concentrations of tricaine from 300 μM to 1 mM. After each solution change, the embryo was allowed to equilibrate with the drug for three minutes prior to imaging.

### Stroke volume, heart rate, and cardiac output calculations

Borrowing principles from human ultrasonic Doppler Imaging, stroke volume and cardiac output were extracted from linescans of the dorsal aorta close to the heart, where there is little influence from peripheral vessels. Stroke volume (SV), the volume of blood ejected by the ventricle in one heartbeat, was determined by multiplying the cross sectional area of the aorta (*A*_*aorta*._) by the distance blood is pumped along the aorta in one beat (*dx*) (Equation 1).

*SV *= *A*_aorta _× *dx*

Assuming a circular dorsal aorta, the cross-sectional area is given by Equation 2, where the diameter of the aorta (*d*_*aorta*._*) *was measured from the x-dimension of the perpendicular linescan.

Aaorta=π(daorta2)2
 MathType@MTEF@5@5@+=feaafiart1ev1aaatCvAUfKttLearuWrP9MDH5MBPbIqV92AaeXatLxBI9gBaebbnrfifHhDYfgasaacH8akY=wiFfYdH8Gipec8Eeeu0xXdbba9frFj0=OqFfea0dXdd9vqai=hGuQ8kuc9pgc9s8qqaq=dirpe0xb9q8qiLsFr0=vr0=vr0dc8meaabaqaciaacaGaaeqabaqabeGadaaakeaacqWGbbqqdaWgaaWcbaGaemyyaeMaem4Ba8MaemOCaiNaemiDaqNaemyyaegabeaakiabg2da9GGaciab=b8aWnaabmaabaWaaSaaaeaacqWGKbazdaWgaaWcbaGaemyyaeMaem4Ba8MaemOCaiNaemiDaqNaemyyaegabeaaaOqaaiabikdaYaaaaiaawIcacaGLPaaadaahaaWcbeqaaiabikdaYaaaaaa@439E@

Because velocity is defined as distance travelled per unit time, integrating cell velocities as a function of time gives the distance travelled (Equation 3).

dx=∫0t′v(t)dt
 MathType@MTEF@5@5@+=feaafiart1ev1aaatCvAUfKttLearuWrP9MDH5MBPbIqV92AaeXatLxBI9gBaebbnrfifHhDYfgasaacH8akY=wiFfYdH8Gipec8Eeeu0xXdbba9frFj0=OqFfea0dXdd9vqai=hGuQ8kuc9pgc9s8qqaq=dirpe0xb9q8qiLsFr0=vr0=vr0dc8meaabaqaciaacaGaaeqabaqabeGadaaakeaacqWGKbazcqWG4baEcqGH9aqpdaWdXaqaaiabdAha2jabcIcaOiabdsha0jabcMcaPiabdsgaKjabdsha0bWcbaGaeGimaadabaGafmiDaqNbauaaa0Gaey4kIipaaaa@3C6A@

Substitution of Equations 2 and 3 into equation 1 gives the final equation for stroke volume (Equation 4).

SV=π(daorta2)2×∫0t′v(t)dt
 MathType@MTEF@5@5@+=feaafiart1ev1aaatCvAUfKttLearuWrP9MDH5MBPbIqV92AaeXatLxBI9gBaebbnrfifHhDYfgasaacH8akY=wiFfYdH8Gipec8Eeeu0xXdbba9frFj0=OqFfea0dXdd9vqai=hGuQ8kuc9pgc9s8qqaq=dirpe0xb9q8qiLsFr0=vr0=vr0dc8meaabaqaciaacaGaaeqabaqabeGadaaakeaacqWGtbWucqWGwbGvcqGH9aqpiiGacqWFapaCdaqadaqaamaalaaabaGaemizaq2aaSbaaSqaaiabdggaHjabd+gaVjabdkhaYjabdsha0jabdggaHbqabaaakeaacqaIYaGmaaaacaGLOaGaayzkaaWaaWbaaSqabeaacqaIYaGmaaGccqGHxdaTdaWdXaqaaiabdAha2jabcIcaOiabdsha0jabcMcaPiabdsgaKjabdsha0bWcbaGaeGimaadabaGafmiDaqNbauaaa0Gaey4kIipaaaa@4BF5@

The integral of *v(t)dt *was approximated by calculating the area under the curve of a plot of velocity as a function of time for each heart beat using Origin v.6.0 (Microcal). Cardiac output, the volume of blood ejected by the heart in one minute, was calculated by multiplying the stroke volume by the heart rate (Equation 5).

*CO *= *SV *× *HR *

Heart rates were determined by dividing 60 seconds by the average duration of a heart beat, which was measured by the average peak-to-peak distance of the velocity-versus-time profile.

### Morpholino knockdown of OSM

Morpholino oligonucleotides targeting the translation initiation site of the *Danio rerio ccm2/osm *mRNA were purchased from Gene Tools, Inc. Two OSM-specific morpholinos were used (MO#1: ATCTAATACAGCGAAAATGAAGAGC; MO#2: ATTTGTACGTAGAGATGGAGGAGGA). Morpholino sequences were designed from the +75 to -25 bp region surrounding the initiating ATG of *Danio rerio ccm2/osm *cDNA (IMAGE Clone 7396516) by Gene Tools, Inc. A non-targeting morpholino or 1× Danieau solution (57 mM NaCl, 0.7 mM KCl, 0.4 mM MgSO4, 0.6 mM Ca(NO3)2, 5 mM HEPES pH 7.6) were used as controls. Unless otherwise specified, morpholinos were dissolved to 2 ng/nl in 1× Danieau solution. Approximately 5 nl of morpholino solution were injected into embryos between the 1- and 4-cell stage. Repression of OSM protein was confirmed by immunoblotting using a rabbit polyclonal anti-OSM antibody produced against recombinant full-length mouse OSM [33].

### Capped OSM mRNA synthesis and rescue

Capped, in vitro transcribed *ccm2/osm *mRNA was synthesized using Mmessage Mmachine T7 kit (Ambion) from the coding region of a *Danio rerio *OSM cDNA (IMAGE Clone 7396516). Embryos between the 1- and 4-cell stages were injected with 5 nl *ccm2/osm *mRNA (20–100 pg/nl) together with an OSM morpholino that targets the 5' UTR (MO#1) at 2–4 ng/nl).

### *In silico *microangiography

In *silico *microangiography is an image processing approach to representing motion in a series of two-dimensional images. A DIC movie of cells flowing through the vasculature of anesthetized embryos at 28 or 48 hpf was collected as a set of 100 12-bit tiff images recorded at 15 ms intervals (X, Y)._t_. Using Metamorph IP software (Molecular Devices) a difference image stack was created by dividing adjacent time points; that is, for time t = n, Difference._n _= (X, Y)._n+1. _*1000/(X, Y)._n._. The resultant difference stack has intensity values only where changes occurred from one time point to the next. Maximum intensity projections through the differences stack produce an image correlating to blood cell movement over the duration of the original movie.

## Authors' contributions

MHM and NS conceived of the study. MHM performed zebrafish husbandry, morpholino knockdown and drug treatment experiments, acquired images, wrote the laser-scanning velocimetry data analyzer software, analyzed linescan images and drafted the manuscript. NS developed image acquisition protocols, processed images for linescans, performed *in silico *microangiography, and assisted in drafting the manuscript. LS performed zebrafish husbandry and morpholino knockdown experiments. KH assisted in developing image acquisition protocols. EL assisted with zebrafish husbandry and morpholino knockdown experiments. GLJ participated in the design of the study, coordinated essential collaborations, and assisted in drafting the manuscript. All authors read and approved the final manuscript.

## Supplementary Material

Additional File 1 **position effects**. Dependence of cardiac performance measurements on scan line position Click here for file

Additional File 2 **cardiovascular analysis of OSM morphant**. Decreased circulation in OSM-deficient embryos only affects the aortic arch Click here for file

Additional File 3 **restricted circulation movie**. Restricted blood flow through constricted aortic arch of control and OSM morpholino-injected embryo Click here for file
