# Supplementary material for: Laser-scanning velocimetry: A confocal microscopy method for quantitative measurement of cardiovascular performance in zebrafish embryos and larvae
Source: BMC Biotechnol. 2007 Jul 10;7:40. doi: 10.1186/1472-6750-7-40 (PMC1955438; doi:10.1186/1472-6750-7-40)
Supplement: Additional File 1 — position effects. Dependence of cardiac performance measurements on scan line position [file 1472-6750-7-40-S1.pdf]

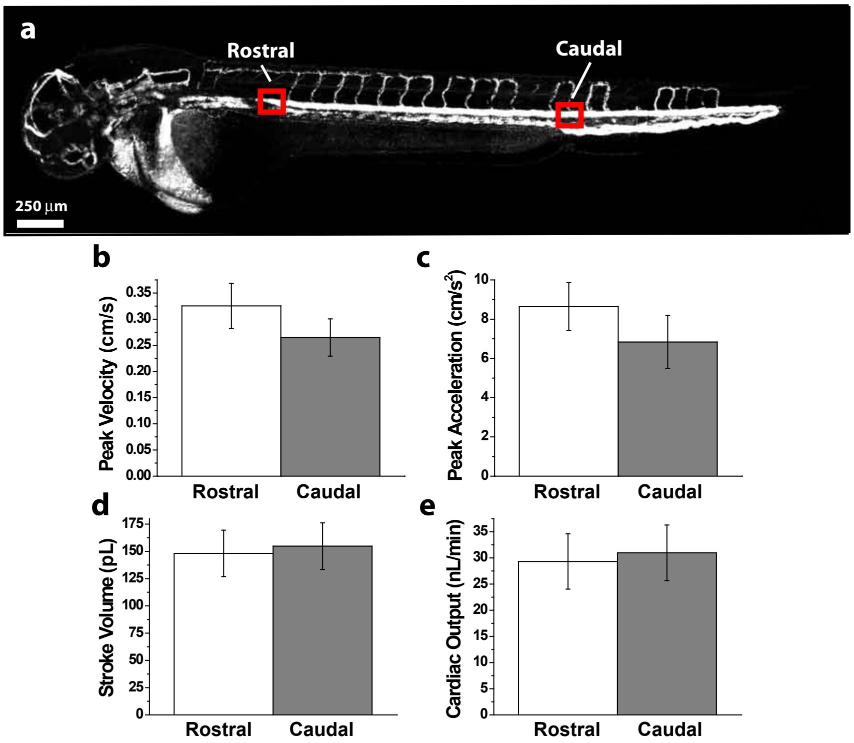

Supplementary Figure 1: Comparison of cardiac performance measurements obtained at a rostral and caudal position along the dorsal aorta of a 48 hpf embryo. a) In silico microangiogram of a 48 hpf zebrafish embryo showing the location of two scan lines used to acquire velocimetry data from a rostral and caudal location along the dorsal aorta. b-e) Peak velocity (b), peak acceleration (c), stroke volume (d), and cardiac output (e) obtained from rostral and caudal scan lines. Error bars represent the SEM, n=3.
