# Supplementary material for: Laser-scanning velocimetry: A confocal microscopy method for quantitative measurement of cardiovascular performance in zebrafish embryos and larvae
Source: BMC Biotechnol. 2007 Jul 10;7:40. doi: 10.1186/1472-6750-7-40 (PMC1955438; doi:10.1186/1472-6750-7-40)
Supplement: Additional File 2 — cardiovascular analysis of OSM morphant. Decreased circulation in OSM-deficient embryos only affects the aortic arch [file 1472-6750-7-40-S2.pdf]

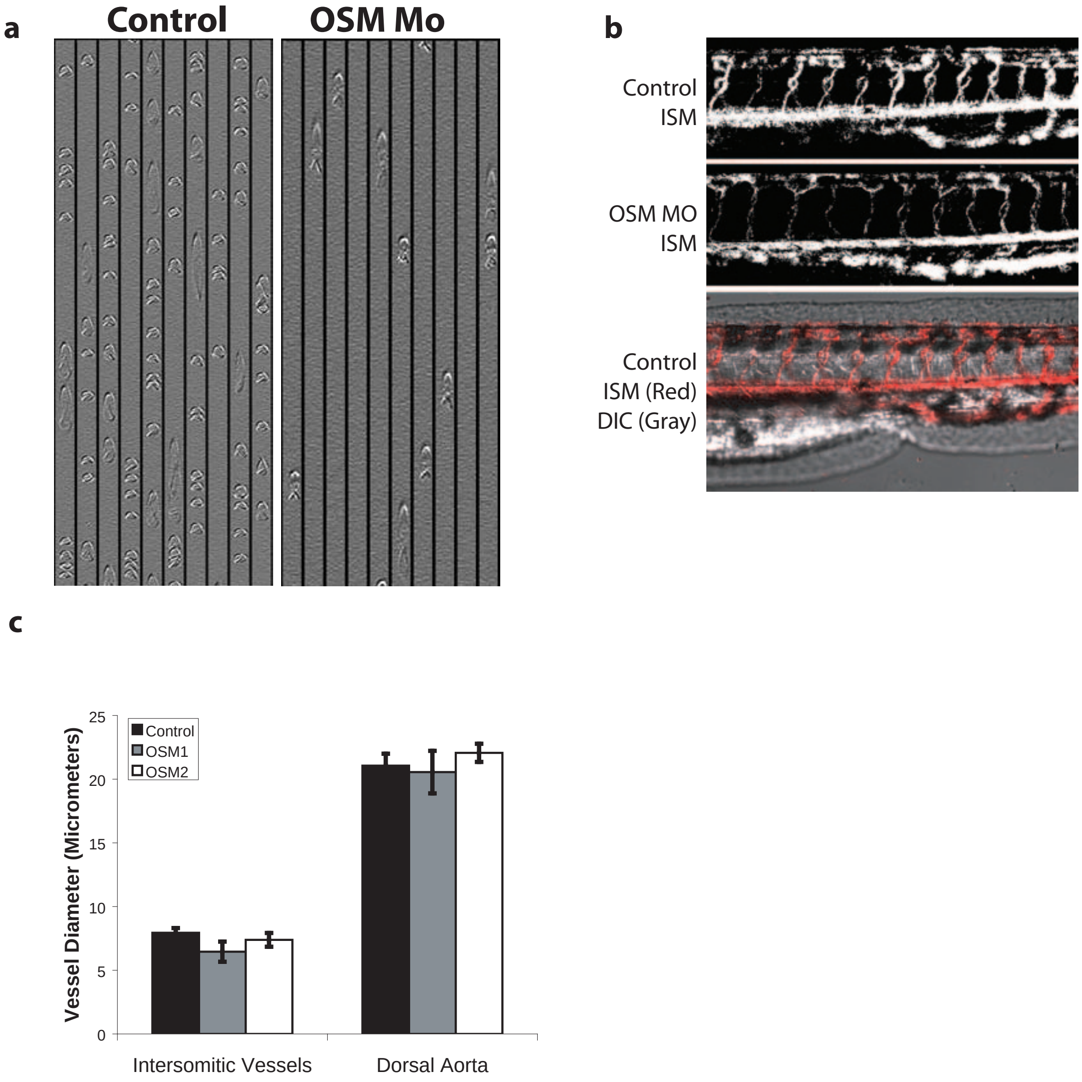

Supplementary Figure 2 - Decreased circulation in OSM-deficient embryos only affects the lumen of the aortic arch

Perpendicular line scan images from the intersomitic vessels of control and OSM-morphant embryos clearly illustrate the decreased circulation observed in OSM morphant embryos at 48 hpf (Supplementary Fig. 1a). In silico microangiograms from the tails of 48 hpf control or OSM-deficient embryos show that the dorsal aorta, formed through vasculogenesis, and the intersomitic vessels, which are formed through angiogenesis, are well developed, properly oriented, and make the appropriate connections in both control and OSM morphant embryos (Supplementary Fig. 1b). Because in silico microangiography requires blood cell motion to delineate the path of a vessel, the intensity of the in silico microangiograms from OSM-deficient embryos were less intense than control embryos. To ensure the decreased intensity was not the result of decreased vessel diameters, the diameter of intersomitic vessels and the dorsal aorta were measured by DIC microscopy. Diameters were not different in control embryos of those injected with either of two OSM morpholinos (Supplementary Fig. 1c).
